# Assessment of *HMGA2* and *PLAG1* rearrangements in breast adenomyoepitheliomas

**DOI:** 10.1038/s41523-018-0101-7

**Published:** 2019-01-18

**Authors:** Fresia Pareja, Felipe C. Geyer, David N. Brown, Ana P. Martins Sebastião, Rodrigo Gularte-Mérida, Anqi Li, Marcia Edelweiss, Arnaud Da Cruz Paula, Pier Selenica, Hannah Y. Wen, Achim A. Jungbluth, Zsuzsanna Varga, Juan Palazzo, Brian P. Rubin, Ian O. Ellis, Edi Brogi, Emad A. Rakha, Britta Weigelt, Jorge S. Reis-Filho

**Affiliations:** 1 Department of Pathology, Memorial Sloan Kettering Cancer Center, New York, NY USA; 2 Institute of Surgical Pathology, University Hospital Zurich, Zurich, Switzerland; 3 Department of Pathology, Thomas Jefferson University Hospital, Philadelphia, PA USA; 4 Department of Pathology, Cleveland Clinic, Cleveland, OH USA; 5 Department of Pathology, University of Nottingham, Nottingham, UK

## Abstract

Breast adenomyoepitheliomas (AMEs) are rare epithelial-myoepithelial neoplasms that may occasionally produce myxochondroid matrix, akin to pleomorphic adenomas (PAs). Regardless of their anatomic location, PAs often harbor rearrangements involving *HMGA2* or *PLAG1*. We have recently shown that the repertoire of somatic genetic alterations of AMEs varies according to their estrogen receptor (ER) status; whilst the majority of ER-positive AMEs display mutually exclusive *PIK3CA* or *AKT1* hotspot mutations, up to 60% of ER-negative AMEs harbor concurrent *HRAS* Q61 hotspot mutations and mutations affecting either *PIK3CA* or *PIK3R1*. Here, we hypothesized that a subset of AMEs lacking these somatic genetic alterations could be underpinned by oncogenic fusion genes, in particular those involving *HMGA2* or *PLAG1*. Therefore, we subjected 13 AMEs to RNA-sequencing for fusion discovery (*n* = 5) and/or fluorescence in situ hybridization (FISH) analysis for *HMGA2* and *PLAG1* rearrangements (*n* = 13). RNA-sequencing revealed an *HMGA2*-*WIF1* fusion gene in an ER-positive AME lacking *HRAS, PIK3CA* and *AKT1* somatic mutations. This fusion gene, which has been previously described in salivary gland PAs, results in a chimeric transcript composed of exons 1–5 of *HMGA2* and exons 3–10 of *WIF1*. No additional in-frame fusion genes or *HMGA2* or *PLAG1* rearrangements were identified in the remaining AMEs analyzed. Our results demonstrate that a subset of AMEs lacking mutations affecting *HRAS* and PI3K pathway-related genes may harbor *HMGA2*-*WIF1* fusion genes, suggesting that a subset of breast AMEs may be genetically related to PAs or that a subset of AMEs may originate in the context of a PA.

## Genetics: uncommon breast tumors linked genetically to salivary gland cancer

A subset of benign breast tumors known as adenomyoepitheliomas may be driven by gene fusions found in related cancers elsewhere in the body. Adenomyoepitheliomas are rare benign tumors characterized by the proliferation of both luminal epithelial cells and adjacent myoepithelial cells. Since these tumors resemble a common type of salivary gland cancer called pleomorphic adenoma that often harbors genomic rearrangements involving two particular genes, Jorge Reis-Filho from Memorial Sloan Kettering Cancer Center in New York City, USA, and colleagues looked for similar oncogenic fusions in 13 breast adenomyoepitheliomas with no known causative genetic alterations. They identified a gene fusion in one of their 13 tumor samples, suggesting that a subset of adenomyoepitheliomas may be genetically related to pleomorphic adenomas. The findings could aid in future treatment of both diseases.

## Introduction

Breast adenomyoepitheliomas (AMEs) are rare neoplasms with dual epithelial-myoepithelial differentiation,^1^ composed of gland-like structures containing an inner layer of pink, eosinophilic epithelial cells and an abluminal layer of often clear, myoepithelial cells. AMEs can display a variety of histologic appearances, and be either estrogen receptor (ER)-positive or ER-negative.^1,2^ Although there is overlap in the histologic features of ER-positive and ER-negative AMEs, we have recently shown that the repertoire of genetic alterations of these tumors vary according to their ER status.^3^ Whilst ER-negative AMEs harbor *HRAS* Q61 hotspot mutations co-occurring with mutations affecting *PIK3CA* or *PIK3R1* in up to 60% of cases, the majority of ER-positive AMEs were found to display seemingly mutually exclusive *PIK3CA* or *AKT1* activating hotspot mutations.^3^

In the spectrum of histologic appearances of AMEs, myxochondroid matrix has been occasionally described.^4^ This type of matrix bears histologic resemblance to the matrix of pleomorphic adenomas (PAs),^1^ epithelial-myoepithelial neoplasms that may arise in various anatomic locations, including the breast.^5^ PAs are underpinned by recurrent gene rearrangements involving *HMGA2* or *PLAG1* in up to 65% of cases, regardless of their anatomic origin.^6–8^ Due to the overlapping histologic appearances of AMEs and PAs, we sought to determine whether a subset of AMEs, primarily those lacking mutations affecting known drivers (e.g., *HRAS* or PI3K pathway-related genes), would be genetically related to PAs, and would be underpinned by fusion genes, in particular those involving *HMGA2* and *PLAG1*.

## Results

### *HMGA2-WIF1* fusion gene in an ER-positive AME

**Fig. 1:**
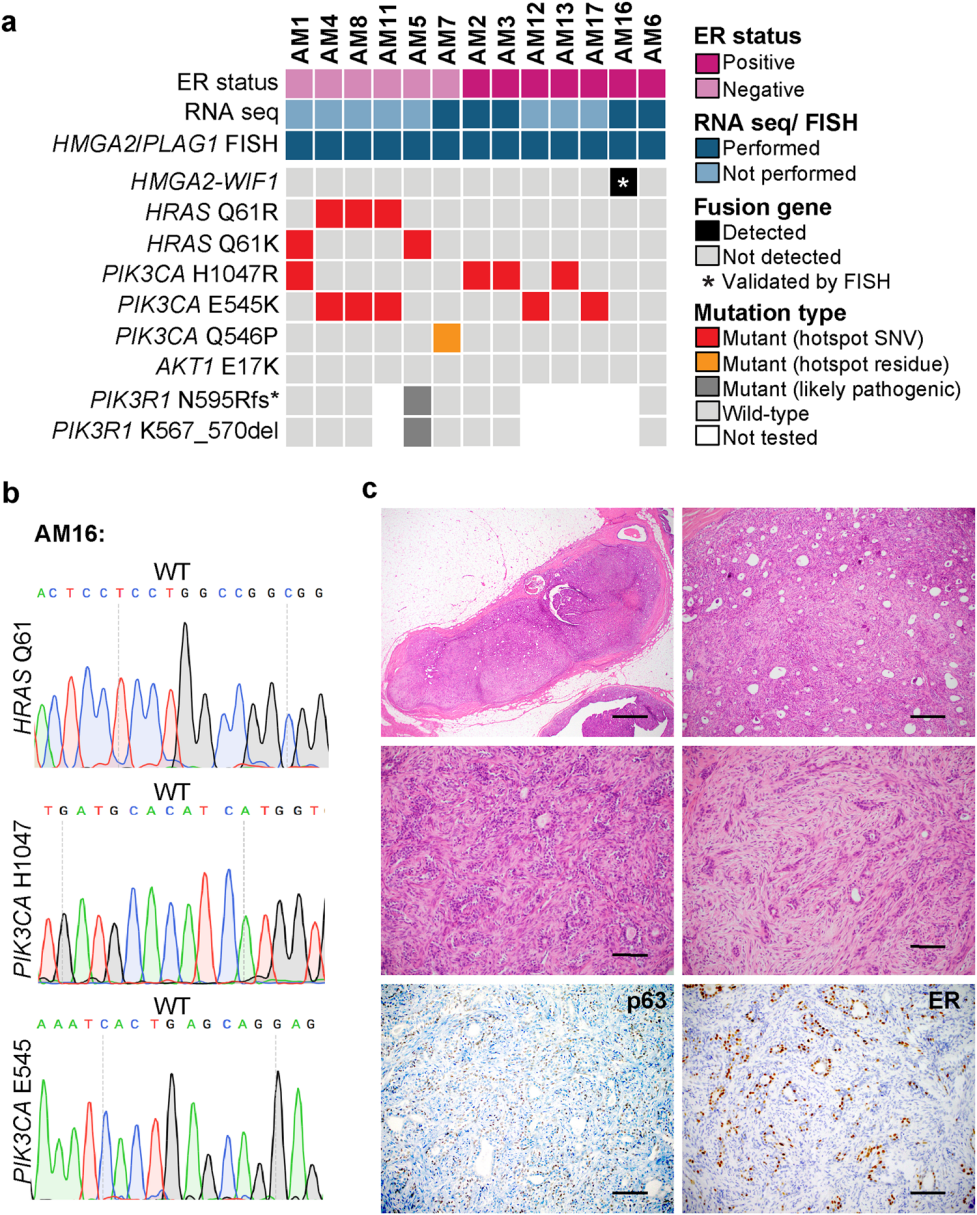
Fusion genes involving *HMGA2* or *PLAG1* and somatic mutations targeting *HRAS*, *PIK3CA, AKT1* and *PIK3R1* in breast adenomyoepitheliomas. **a** Heatmap depicting fusion gene and somatic mutations targeting *HRAS* Q61, *PIK3CA* and *AKT* E17 hotspot loci and *PIK3R1* mutations identified in breast adenomyoepitheliomas (AMEs; *n* = 13). Cases are shown in columns and genes in rows. Hotspot mutations are annotated as per Chang et al.^23^ **b** Representative Sanger sequencing electropherograms of *HRAS* Q61 and *PIK3CA* hotspot loci in AM16. **c** Representative hematoxylin and eosin micrographs of an AME harboring an *HMGA2*-*WIF1* fusion gene (AM16), and micrographs depicting p63 and estrogen receptor expression. Scale bars, 500 μm (upper left), 100 μm (upper right) and 50 μm (middle and lower panels). *ER* estrogen receptor, *FISH* fluorescence in situ hybridization, *SNV* single nucleotide variant, *WT* wild-type

Thirteen breast AMEs, whose whole-exome, targeted capture and/or Sanger sequencing and ER status were previously described in Geyer et al,^3^ were included in this study (Table 1). Six cases were ER-negative and seven were ER-positive. Four ER-negative AMEs harbored concurrent *PIK3CA* and *HRAS* mutations (4/6), one harbored an *HRAS* Q61K mutation and concurrent likely pathogenic *PIK3R1* mutations (1/6), and one was *HRAS* wild-type and harbored a *PIK3CA* mutation (1/6). Five ER-positive AMEs harbored *PIK3CA* mutations (5/7), and all were wild-type for *HRAS*. None of the cases harbored mutations affecting the *AKT1* E17 hotspot locus (Fig. 1a and Supplementary Fig. 1). Notably, all *HRAS* and *PIK3CA* mutations were classical activating hotspot mutations, except for one *PIK3CA* mutation (Q546) which targeted a hotspot residue and was predicted to be likely pathogenic (Fig. 1a).

**Table 1:** Clinicopathological and selected genetic features of the 13 breast adenomyoepitheliomas included in this study

Case ID	ER status	*PIK3CA* status	*AKT1* status	*PIK3R1* status	*HRAS* status	Oncogenic fusion genes by RNA sequencing	*PLAG1*/ *HMGA2* rearrangement by FISH
AM1	Negative	MUT	WT	WT	MUT	NT	Negative
AM4	Negative	MUT	WT	WT	MUT	NT	Negative
AM8	Negative	MUT	WT	WT	MUT	NT	Negative
AM11	Negative	MUT	WT	NT	MUT	NT	Negative
AM5	Negative	WT	WT	MUT	MUT	NT	Negative
AM7	Negative	MUT	WT	WT	WT	None	Negative
AM2	Positive	MUT	WT	WT	WT	None	Negative
AM3	Positive	MUT	WT	WT	WT	None	Negative
AM12	Positive	MUT	WT	NT	WT	NT	Negative
AM13	Positive	MUT	WT	NT	WT	NT	Negative
AM17	Positive	MUT	WT	NT	WT	NT	Negative
AM16	Positive	WT	WT	NT	WT	*HMGA2-WIF1*	Positive (*HMGA2*)
AM6	Positive	WT	WT	WT	WT	None	Negative

*ER* estrogen receptor, *MUT* mutant, *NT* not tested, *WT* wild-type

To determine whether AMEs lacking *HRAS* Q61 hotspot mutations would harbor fusion genes, we subjected five *HRAS* wild-type AMEs with available material to RNA-sequencing analysis for an unbiased detection of expressed fusion genes (Supplementary Fig. 1). Using a validated pipeline for the *de novo* discovery of fusion genes,^9^ we identified an *HMGA2*-*WIF1* fusion gene in an ER-positive *HRAS-*/*PIK3CA-*/*AKT1*-wild-type AME (AM16) (Figs. 1a-c, Table 1 and Supplementary Table 1). The *HMGA2*-*WIF1* fusion gene identified in AM16 results in a chimeric transcript encompassing all five exons and the initial segment of the 3′ UTR of *HMGA2* fused to exons 3–10 of *WIF1*, and is predicted to be translated to a full length HMGA2 protein and an N-terminal truncated WIF1 protein, with a truncated WIF domain. *WIF1* encodes for a tumor suppressor that modulates Wnt signaling, a role that requires an intact WIF domain.^10^

The AME found to harbor the *HMGA2*-*WIF1* fusion gene (AM16) displayed the typical histologic features of AMEs,^3^ and constituted a well-circumscribed lesion with pushing borders, surrounded by a thick fibrous capsule. This lesion displayed a nodular architecture and a mixed tubular and papillary growth pattern. No cellular atypia, mitotic activity or necrosis was identified (Fig. 1c). Focal areas with conspicuous stroma with myxoid quality were observed (Fig. 1c). The myoepithelial component was highlighted by p63 on immunohistochemical analysis, and strong ER expression was observed in the epithelial component (Fig. 1c).

Given that in the salivary glands, epithelial-myoepithelial carcinomas, the salivary gland counterpart of breast AME, can occasionally originate in the context of a PA (i.e., the so-called carcinoma ex-PA),^11^ we sought to define if AM16 would have areas diagnostic of PA. An independent pathology review of all slides available from this AME by five pathologists failed to reveal any areas that would be consistent with a diagnosis of PA.

### Breast AMEs lack recurrent *HMGA2* or *PLAG1* rearrangements

None of the additional AMEs subjected to RNA-sequencing harbored other fusion genes involving gene partners previously described in PAs,^12^ in myoepitheliomas of other anatomical sites (i.e., *EWSR1* and *FUS* rearrangements),^13,14^ or in other tumors displaying myoepithelial differentiation (i.e., *CRTC1*-*MAML2* fusion gene in mucoepidermoid carcinomas or *MYB* and *MYBL1* rearrangements in adenoid cystic carcinoma).^9,15^ No additional likely pathogenic in-frame fusion gene was identified in the cases subjected to RNA-sequencing analysis (Supplementary Table 1).

**Fig. 2:**
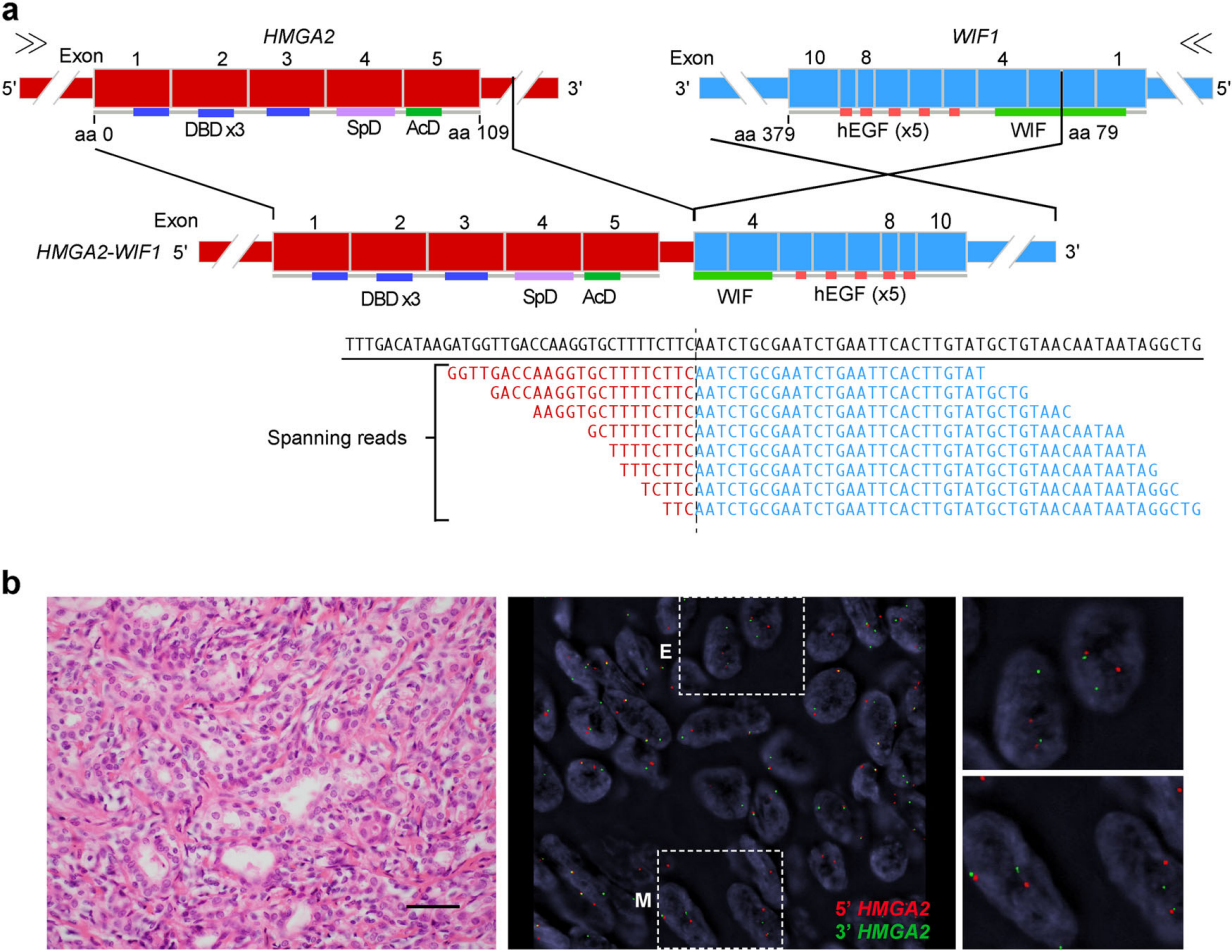
*HMGA2*-*WIF1* fusion gene identified in the epithelial and myoepithelial cells of a breast adenomyoepithelioma. **a** Schematic representation of the *HMGA2*-*WIF1* fusion transcript identified in AM16, including the exons and domains involved. *HMGA2* is on the (+) DNA strand and *WIF1* on the (−) DNA strand. The breakpoints of the 5’ and 3’ partner genes are represented as black vertical lines. Eight spanning reads were found to cross the genomic breakpoint of the *HMGA2*-*WIF1* chimeric transcript and are depicted aligned to the predicted junction sequence. **b** Representative hematoxylin and eosin and FISH micrographs of the epithelial and myoepithelial components of AM16 using *HMGA2* dual-color break apart probes (red, 5′ *HMGA2*; green, 3′ *HMGA2*). aa aminoacid, *AcD* acidic domain, *DBD* DNA binding domain, *E* epithelium, *M* myoepithelium, *SpD* spacer domain. Scale bar, 50 μm

Given that *HMGA2* and *PLAG1* rearrangements have been described in other neoplasms with epithelial-myoepithelial differentiation, in particular in PAs, we sought to define whether AMEs may harbor fusion genes known to underpin PAs. We subjected the five AMEs analyzed by RNA-sequencing and all the other AMEs included in this study (n = 8) to FISH using *HMGA2* and *PLAG1* dual-color break apart probes (Table 1 and Supplementary Fig. 1). This analysis confirmed the presence of an *HMGA2* rearrangement in both the epithelial and myoepithelial components of AM16 (Fig. 2b) and did not reveal any additional AMEs harboring *HMGA2* or *PLAG1* rearrangements (Table 1).

## Discussion

We have previously shown that approximately 60% ER-positive AMEs harbor *PIK3CA* or *AKT1* mutations, whereas up to 60% of ER-negative AMEs are characterized by *HRAS* Q61 mutations concurrent with mutations affecting genes of the PI3K signaling pathway.^3^ Given the occasional histologic similarities between AMEs and PAs, and the fact that a subset of AMEs lack a known driver genetic alteration in the form of somatic mutations affecting protein coding genes, we posited that a subset of AMEs may harbor oncogenic fusion genes previously described in other myoepithelial lesions including PAs. Our analyses resulted in the identification of an ER-positive *HRAS-*/*PIK3CA-*/*AKT1*-wild type AME harboring an *HMGA2*-*WIF1* fusion gene, which has been described in PAs and carcinomas ex-PA of the salivary gland.^10,16^

The high-mobility group AT-hook 2 (*HMGA2*) gene encodes for a transcriptional regulator of genes involved in cell proliferation and cell death.^17^ HMGA2 overexpression plays a key role in oncogenic transformation through several mechanisms, such as the induction of E2F1 and AP1 activity, promotion of cyclin A expression, inactivation of p53-dependent apoptosis, and activation of the TGF-β signaling pathway.^17,18^ The Wnt signaling pathway is regulated by secreted antagonists that bind to Wnt proteins, preventing ligand-receptor interactions,^19^ such as WIF1.^20^ WIF1 consists of an N-terminal secretion signal, five EGF-like domains, a hydrophilic C-terminus, and a WIF domain, which is required for binding to Wnt proteins and for the tumor suppressor properties of *WIF1*.^19^ The *HMGA2*-*WIF1* fusion gene identified in AM16 has been shown to result in increased HMGA2 expression, presumably due to loss of regulatory sites in its 3′ UTR,^21^ and decreased WIF1 expression.^10^ The *HMGA2*-*WIF1* chimeric transcript identified in our study is predicted to encode a full length HMGA2 protein and an N-terminally truncated WIF1 protein harboring a truncated WIF domain. Given that the *HMGA2* breakpoint maps to its 3’ UTR after the stop codon, it is possible that *WIF1* may not even be translated, akin to a rearrangement involving the same *HMGA2* and *WIF1* exons previously described in a PA arising in the salivary gland.^16^ In addition, *HMGA2* and *WIF1* display opposite transcriptional orientations, and this fusion gene may stem from a cryptic paracentric inversion (Fig. 2a).^16^

Taken together, the *HMGA2*-*WIF1* fusion identified here might result in increased expression of *HMGA2*, with ensuing activation of TGF-β signaling, along with derepression of Wnt signaling. Taken together, our findings suggest that a subset of AMEs lacking genetic alterations involving genes of the RAS-MAPK pathway may be underpinned by fusion genes resulting in the activation of alternative signaling pathways, such as TGF-β and Wnt.

One could posit that the AME harboring the *HMGA2*-*WIF1* fusion gene described in this study would, in fact, constitute a breast PA. This case was independently reviewed by five breast pathologists who concurred in the diagnosis of AME. It should be noted, however, that despite being a *bona fide* AME, this case focally displayed increased myxoid stroma, bearing some resemblance to, but not fulfilling the diagnostic criteria for, a breast PA. Another potential explanation for the presence of this fusion gene in AM16 is that it would constitute the breast equivalent of the salivary gland epithelial-myoepithelial carcinoma ex-PA.^11^ The central pathology review of all slides available from this case failed to reveal any areas diagnostic of PA. Therefore, our findings suggest that a subset of AMEs share not only morphologic features with PAs, but may also resemble PAs at the genetic level. We cannot rule out, however, that AM16 developed in the context of a PA, which was subsequently obliterated by the outgrowth of the AME.

The FISH analysis of AM16 revealed the presence of the *HMGA2*-*WIF1* both in the epithelial and myoepithelial cells of the tumor (Fig. 2b). This observation is consistent with the notion that in AMEs, both the epithelial and myoepithelial components are neoplastic and clonally related, even though in this AME (AM16), the epithelial component was ER-positive, whereas the myoepithelial component was ER-negative.

Our study was several limitations, such as the small size of our cohort and the fact that we included only two *HRAS-*/*PIK3CA*-/*AKT1*-wild type AMEs, given that no additional material from other AMEs was available for transcriptomic or FISH analysis. The limited sample size of our study precludes definitive conclusions regarding the relationship between ER status and the presence of the *HMGA2-WIF1* fusion gene in AMEs to be drawn. Despite these limitations, our findings demonstrate that AMEs lacking mutations affecting *HRAS, PIK3CA* and *AKT1* may harbor the *HMGA2*-*WIF1* fusion gene, previously described in salivary gland PAs and carcinomas ex-PA,^10,16^ suggesting that a subset of AMEs may be genetically related to PAs or that AMEs may originate in the context of breast PAs.

## Methods

### Cases and DNA sequencing data

This study was approved by the Institutional Review Boards (IRBs) and local research ethics committees of the authors’ institutions. Patient consent was obtained if required by the approved IRB protocols. In this study we included thirteen breast AMEs, retrieved from the authors’ institutions and previously described by Geyer et al.^3^ Whole-exome sequencing, MSK-IMPACT and Sanger sequencing data, and immunohistochemical data were retrieved from Geyer et al.^3^ ER status was assessed by immunohistochemistry according to the current ASCO/CAP guidelines.^22^ Hotspot mutations are annotated as per Chang et al.^23^ For power calculations, if we posited that an *HRAS*-wild type AMEs would be underpinned by a recurrent fusion gene and that this fusion gene would be present in ≥ 70% of cases akin to recurrent fusion genes in other tumor types,^9,24–26^ sequencing analysis of five samples would confer 80% power for its detection.

### RNA-sequencing and the identification of fusion transcripts

RNA-sequencing was performed on five *HRAS*-wild type AMEs according to standard protocols employed at the Integrated Genomics Operation of Memorial Sloan Kettering Cancer Center (MSKCC).^27^ In brief, paired-end massively parallel RNA-sequencing (2 × 50 bp) was performed on a HiSeq2000 (Illumina), as previously described.^28^ Read pairs supporting chimeric transcripts were identified using deFuse,^29^ and INTEGRATE,^30^ followed by exclusion of candidate fusion transcripts found in a set of 287 normal breast tissues from the TCGA dataset,^31^ as previously described.^28^ The Bayesian probability of the remaining candidate fusion genes, supported by at least two spanning reads, to constitute drivers was annotated using OncoFuse,^32^ as previously described.^28^

### Fluorescence in situ hybridization (FISH)

All cases included in this study (*n* = 13) were subjected to FISH analysis for *HMGA2* and *PLGA1* using dual-color break-apart probes following validated protocols at the MSKCC Molecular Cytogenetics Core, as previously described.^33^ The probe mix consisted of bacterial artificial chromosome (BAC) clones mapping to 5′ *HMGA2* (RP11-230G5, RP11-662G15; red) and 3′ *HMGA2* (RP11-937C6, RP11-167E10; green), and BAC clones mapping to 5′ *PLAG1* (RP11-92A9, RP11-111I18; red) and 3′ *PLAG1* (RP11-144E19, RP11-246A9; green). A minimum of 50 interphase nuclei were analyzed for *HMGA2* or *PLAG1* rearrangements. Cases were considered positive for rearrangement if separation of the 5′ (red) and 3′ (green) signals (>2 signal width apart) was identified in >15% tumor cells. FISH analyses were performed with observers blinded to the results of the RNA-sequencing analysis.

## Supplementary information

Supplementary Materials

## Data Availability

RNA-sequencing data that support the findings of this study have been deposited in the NCBI Sequence Read Archive (SRA) under the accession code SRP158271. WES and MSK-IMPACT sequencing data retrieved from Geyer et al^3^ are available in SRA under the accession numbers SRP065277 and SRP065302, respectively. All available data are available from the authors.

## **Supplementary information**

**Supplementary information** accompanies the paper on the *npj Breast Cancer* website (10.1038/s41523-018-0101-7).
