# Supplementary material for: Assessment of HMGA2 and PLAG1 rearrangements in breast adenomyoepitheliomas
Source: NPJ Breast Cancer. 2019 Jan 18;5:6. doi: 10.1038/s41523-018-0101-7 (PMC6338730; doi:10.1038/s41523-018-0101-7)
Supplement: Supplementary file 1 — Supplementary Materials [file 41523_2018_101_MOESM1_ESM.pdf]

# SUPPLEMENTARY MATERIALS

## Assessment of *HMGA2* and *PLAG1* rearrangements in breast adenomyoepitheliomas

Pareja et al.

**Supplementary Figure 1**

**Supplementary Table 1**

**Supplementary Figure 1**

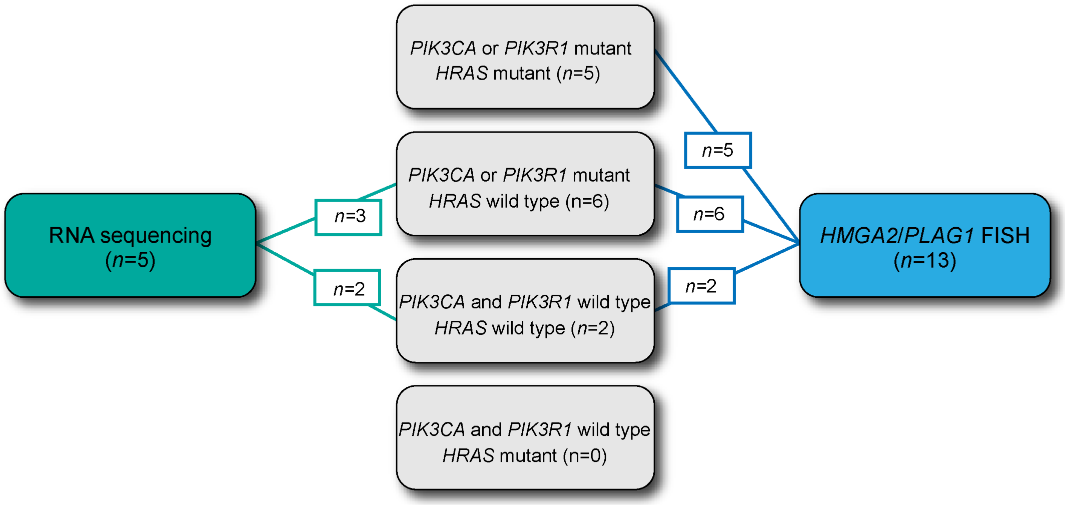


**Supplementary Figure 1: Schematic representation of the cases and analyses conducted in this study.**

Depiction of the *PIK3CA*, *PIK3R1* and *HRAS* status of the breast adenomyoepitheliomas included in this series, and the analyses conducted in this study. FISH, fluorescence *in situ* hybridization.

**Supplementary Table 1: List of fusion genes and/or read-throughs identified by RNA-sequencing analysis of breast adenomyoepitheliomas.**

| Sample ID | Fusion caller* | 5' gene | 3' gene | Mapping 5' | Mapping 3' | Spanning reads | Encompassing reads | In-frame | Type | Driver probability (Oncofuse) | Remarks |
|---|---|---|---|---|---|---|---|---|---|---|---|
| AM16 | INTEGRATE | HMGA2 | WIF1 | chr12:66358254 | chr12:65476942 | 8 | 13 | Yes | Intra-Chromosomal | 0.082892348 | No start codon for 3' partner gene |
| AM2 | INTEGRATE | EEF1A1 | PAN3 | chr6:74229617 | chr13:28788787 | 4 | 3 | Yes | Inter-Chromosomal | 0.288187631 | Intronic breakpoint |
| AM7 | INTEGRATE | RRP7B | KCTD19 | chr22:42961117 | chr16:67355607 | 22 | 8 | No | Inter-Chromosomal | 0.047062962 | |
| AM7 | INTEGRATE | GRM5 | SCARNA9 | chr11:88345973 | chr11:93454952 | 4 | 2 | No | Intra-Chromosomal | 0.021825573 | |
| AM7 | INTEGRATE | GRM5 | KIAA1731 | chr11:88345973 | chr11:93454952 | 3 | 2 | No | Intra-Chromosomal | 0.021825573 | |

*deFuse did not identify any candidates not found in normal breast or that can be mapped to RefSeq genes.
